# Radioluminescence imaging feasibility for robotic radiosurgery field size quality assurance

**DOI:** 10.1002/mp.15914

**Published:** 2022-08-24

**Authors:** Zahra Shakarami, Sara Broggi, Antonella Del Vecchio, Claudio Fiorino, Antonello E. Spinelli

**Affiliations:** ^1^ Experimental Imaging Centre San Raffaele Scientific Institute Via Olgettina 60, 20132 Milan Italy; ^2^ Medical Physics Department San Raffaele Scientific Institute Via Olgettina 60, 20132 Milan Italy

**Keywords:** CyberKnife®, quality assurance, radioluminescence imaging

## Abstract

**Purpose:**

To investigate the feasibility of radioluminescence imaging (RLI) as a novel 2D quality assurance (QA) dosimetry system for CyberKnife®.

**Methods:**

We developed a field size measurement system based on a commercial complementary metal oxide semiconductor (CMOS) camera facing a radioluminescence screen located at the isocenter normal to the beam axis. The radioluminescence light collected by a lens was used to measure 2D dose distributions. An image transformation procedure, based on two reference phantoms, was developed to correct for projective distortion due to the angle (15°) between the optical and beam axis. Dose profiles were measured for field sizes ranging from 5 mm to 60 mm using fixed circular and iris collimators and compared against gafchromic (GC) film. The corresponding full width at half maximum (FWHM) was measured using RLI and benchmarked against GC film. A small shift in the source‐to‐surface distance (SSD) of the measurement plane was intentionally introduced to test the sensitivity of the RLI system to field size variations. To assess reproducibility, the entire RLI procedure was tested by acquiring the 60 mm circle field three times on two consecutive days.

**Results:**

The implemented procedure for perspective image distortion correction showed improvements of up to 1 mm using the star phantom against the square phantom. The FWHM measurements using the RLI system indicated a strong agreement with GC film with maximum absolute difference equal to 0.131 mm for fixed collimators and 0.056 mm for the iris. A 2D analysis of RLI with respect to GC film showed that the differences in the central region are negligible, while small discrepancies are in the penumbra region. Changes in field sizes of 0.2 mm were detectable by RLI. Repeatability measurements of the beam FWHM have shown a standard deviation equal to 0.11 mm.

**Conclusions:**

The first application of a RLI approach for CyberKnife® field size measurement was presented and tested. Results are in agreement with GC film measurements. Spatial resolution and immediate availability of the data indicate that RLI is a feasible technique for robotic radiosurgery QA.

## 1 INTRODUCTION

The CyberKnife® system is a radiotherapy modality that employs multiple narrow beams to deliver conformed and precise radiation dose to the target from different directions in single or few fractions.^1^, ^2^, ^3^, ^4^, ^5^, ^6^ The treatment beam of CyberKnife® is normally shaped using fixed circular, variable aperture iris, and in some cases with multileaf collimators. The circular cones provide circle field diameters ranging from 5 mm to 60 mm. The iris collimator creates fields of polygon with different sizes from 5 mm to 60 mm. Using such small field sizes, accurate and reliable dose measurements methods are required.^7^, ^8^, ^9^, ^10^, ^11^, ^12^, ^13^

Radiochromic films are the gold standard for 2D verification of dose distributions during radiation therapy and for many geometric quality assurance (QA) tests.

Shiomi H et al., calculated the total pointing error of the therapy beam, using GAF MD‐55 radiochromic film before treatment of the patient, aiming at improvement in the accuracy of the CyberKnife® irradiation.^14^ Radiochromic films are almost tissue equivalent and have high spatial resolution, dose rate independence, no angular dependence, and little energy dependence.^15^, ^16^ Although, at the same time, they are expensive, inherently off‐line dosimeters, and it is necessary to wait up to 24 h after film exposure. This makes the use of radiochromic films cumbersome and time consuming.^17^ The spatial resolution of ion chambers and diode arrays is rather low (about few mm) and, thus, cannot be use to perform geometric CyberKnife® QA. Consequently, supplementing or replacing geometric QA measurements using robust, reliable, and repeatable 2D dosimetry system, can be beneficial for CyberKnife® facilities.

**TABLE 1:** FWHM (mm) obtained from the RLI line profiles shown in Figures 5 and 6 and compared with the GC films

	FWHM (mm)		Discrepancy	
Field size (mm)	RLI	GC film	mm	%
*x*‐direction				
Circle 5	6.45	6.50	−0.05	−0.83
Circle 10	10.039	10.108	−0.069	−0.68
Circle 40	41.206	41.269	−0.063	−0.15
Circle 60	61.88	61.82	0.06	0.097
*y*‐direction				
Circle 5	6.601	6.564	0.037	0.56
Circle 10	10.714	10.753	−0.039	−0.36
Circle 40	41.148	41.259	−0.111	−0.26
Circle 60	61.984	61.853	0.131	0.211
Two facing sides				
Iris 5	5.767	5.711	0.056	0.98
Iris 10	9.78	9.80	−0.02	−0.2
Iris 40	37.052	37.095	−0.043	−0.11
Iris 60	56.817	56.866	−0.049	−0.086

**TABLE 2:** The known and measured FWHM (mm) using RLI and GC film. The SSD was shifted on purpose to increase the beam FWHM by 0.2 mm.

Beam size (mm)		Measured FWHM (mm)		Measured diff^a^ (mm)	
SSD	Field	RLI	GC film	RLI	GC film
800	60	61.239	61.656		
802.7	60.2	61.485	61.852	0.246	0.196
800	10	10.466	10.381		
816	10.2	10.783	10.788	0.317	0.407

a Difference.

The use of radioluminescent phosphor‐based radiation visualization technique has made it possible to provide a practical, reliable, and useful tool for radiation beam monitoring and quality control.^18^, ^19^, ^20^, ^21^ This method is known as radioluminescence imaging (RLI) or scintillation imaging and is based on a radioluminescence screen imaged with commercial complementary metal oxide semiconductor (CMOS) or charge coupled device (CCD) camera. The screen is used for the detection and conversion of X‐rays to photons within the visible range. The emitted photons are collected using a lens and detected by a CMOS or CCD camera. The elements composition of the screen should be chosen to provide an high light yield (number of photons generated per unit energy deposited) with a spectrum that matches the peak sensitivity of the camera. RLI images captured by camera may be used for beam monitoring, dose‐distribution verification, and QA purposes in radiation therapy modalities. RLI has been proposed for automating mechanical and geometric tests in routine QA of digital linear accelerator (LINAC).^22^ Furthermore, using the compact hardware design and image processing algorithms, a practical, reliable, and comprehensive solution has been provided based on RLI for high dose rate brachytherapy.^23^ Daniel A. Alexander et al., demonstrated the potential value of scintillation imaging for MR‐linac QA compared with film and comparable sensitivity and linearity to the ion chamber in response to changes in machine output.^24^ RLI has been recently applied also to preclinical small animal radiotherapy for dose distribution verification, showing a good agreement with Monte Carlo dose calculations and GC film measurements for small field of size up to 20× 20 mm^2^.^25^ Besides radiation therapy by photons, the good sensitivity and sub‐mm spatial resolution of RLI system, makes it a useful tool for the quality control of scanning proton beams that allows for the detection of deviations of a few percent in relative dose from the expected dose distribution.^26^ Among advanced radiation therapy techniques, 2D thermoluminescence slab dosimeter coupled with CMOS camera has shown a sufficient precision (<2% beam shape and <3% beam asymmetry) for geometric QA tests and dose‐distribution verification of the CyberKnife®.^27^ While the acquisition time of 200 s and the readout temperature of the heater 400°C result a time‐consuming/lengthy procedure. Therefore, a geometric QA system that is able to obtain accurate field size determination, stable and reproducible results, ease of use (clinical utility), and reasonable measurement time is preferable.

The main goal of this paper was to present, for the first time, the use of RLI as a novel 2D QA dosimetry system for CyberKnife®. An interesting feature of this optical dosimetry system, including the radioluminescence screen coupled with CMOS camera, is the possibility to measure the radiation field in nearly real time, thus avoiding the cumbersome procedures of GF dosimetry. In order to reduce radiation damage to the CMOS and lens both are placed off the X‐rays beam axis, however because of the angle between the optical and the radiation beam main axis, the acquired RLI images are intrinsically distorted; this leads to images with incorrect geometric shape and object dimensions. Thus, image perspective distortion needs to be taken into account in order to recover the geometric features of the radiation field. To this aim, we implemented and improved the homography matrix approach as performed in a previous study by our group focused on preclinical radiotherapy dosimetry.^25^ The perspective distortion approach and its implementation is described in detail in Section 2.1 and will be performed using two reference phantoms. Then, the RLI measurements obtained using different collimators and radiation field sizes are explained in Section 2.2. Extensive comparison of the proposed RLI method will be performed using GC films as gold standard.

## 2 MATERIALS AND METHODS

### 2.1 Perspective image distortion correction

The image perspective distortion correction is based on the use of the plane homography matrix *H*, this method is a standard approach applied in photography.^28^ The homography matrix not only corrects perspective, but also establishes the relationship between pixels and dimensions in the plane of the calibration phantom. The *H* matrix is calculated by matching corresponding points in the reference image $P=\left[\begin{array}{c} X\\ Y\\ 1 \end{array}\right]$, and distorted image $P^{\prime }=\left[\begin{array}{c} X^{\prime }\\ Y^{\prime }\\ 1 \end{array}\right]$:

(1) $$P=HP^{\prime }⟹\left[\begin{array}{c} X\\ Y\\ 1 \end{array}\right]∼\left[\begin{array}{ccc} h_{11} & h_{12} & h_{13}\\ h_{21} & h_{22} & h_{23}\\ h_{31} & h_{32} & h_{33} \end{array}\right]\left[\begin{array}{c} X^{\prime }\\ Y^{\prime }\\ 1 \end{array}\right],$$

where *H* is a 3 × 3 matrix obtained using the projective transformation algorithm implemented in MATLAB R2021a. According to Equations (2) to (4), the homography matrix has 8 degrees of freedom ($h_{33}=1$) and, thus, at least four pairs of corresponding points are necessary to calculate the *H*‐matrix.^29^ By finding the homography *H*, any point in the distorted image space can be mapped to its corresponding point in the undistorted image space.

(2) $$X^{\prime }=\frac{h_{11}X+h_{12}Y+h_{31}}{h_{31}X+h_{32}Y+h_{33}},$$

(3) $$Y^{\prime }=\frac{h_{21}X+h_{22}Y+h_{23}}{h_{31}X+h_{32}Y+h_{33}},$$

(4)

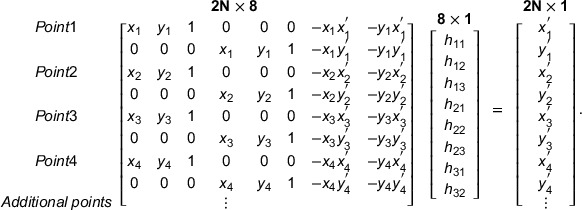




**FIGURE 1:**
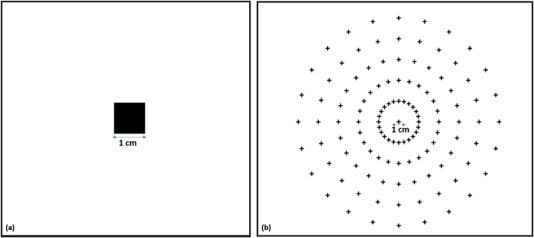
The image shows the two reference phantoms used for perspective image distortion correction; (a) square, (b) star. For the square (10× 10 mm^2^) phantom the transformation matrix *H* was calculated by matching the four corresponding points located at the corners of the reference and measured square phantom images. The star phantom was composed of five concentric circles with 24 cross markers distributed on the circumference of each circle and their diameter increased by 3.5 cm, respectively.

**FIGURE 2:**
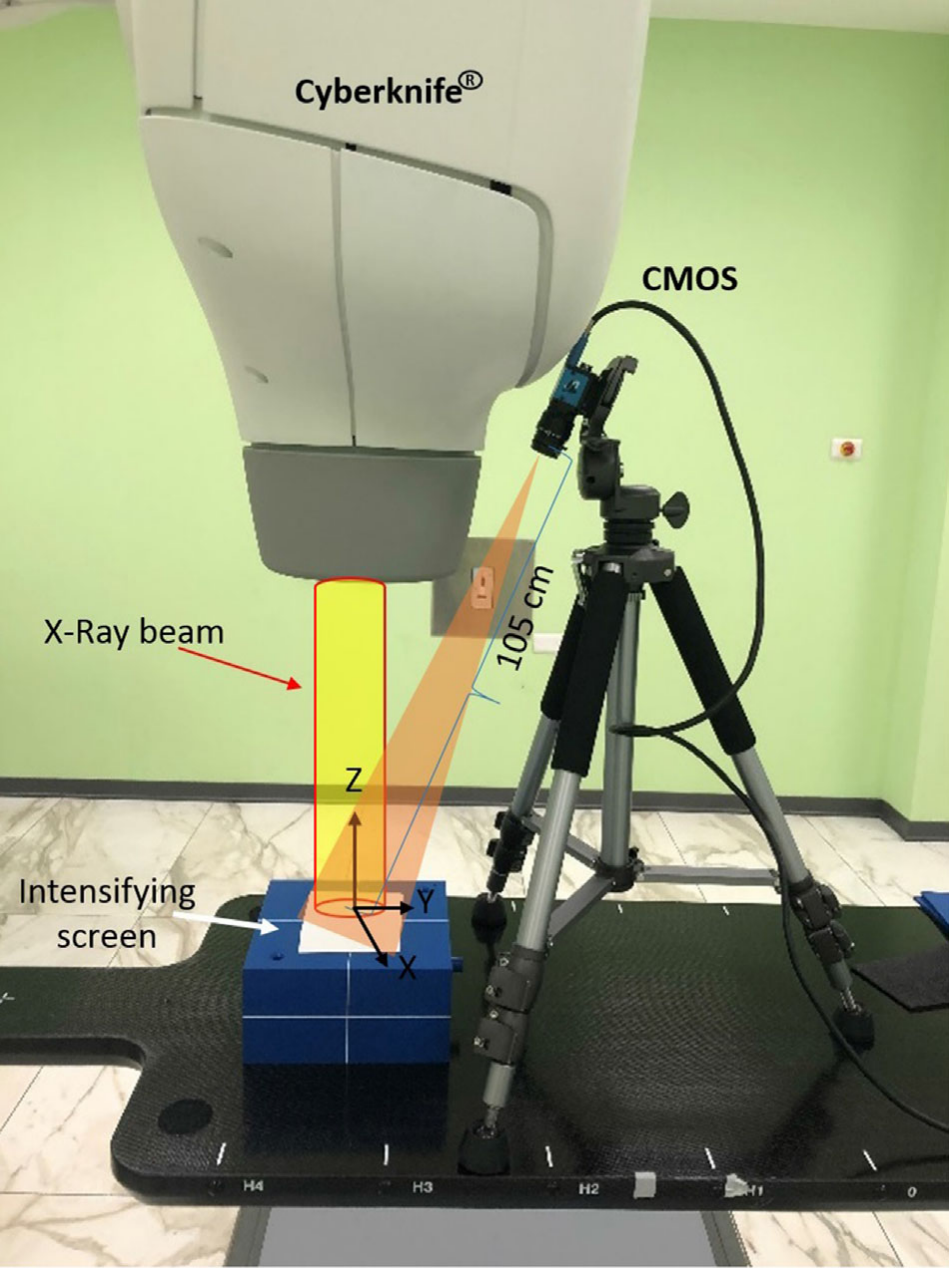
The picture shows the RLI acquisition setup described in Section 2.2. The camera is mounted on a photographic tripod and coupled with an F=1.4, 8‐mm C‐mount lens facing the intensifying screen taped on the stereotactic dose verification phantom. The camera was placed at a distance of 105 cm from the isocenter with an angle between the lens optical axis and vertical of 15°. The (Gd2O2S:Tb) screen was placed at the isocenter, SSD = 80 cm. The radiation beam (yellow) impinged on the scintillation screen, which then emits light collected by the camera (orange).

As will be shown in current work, the accuracy of the *H* matrix calculation and, thus, the image distortion correction can be significantly improved if more reference points are used. In order to investigate the impact on the *H* matrix calculation of the number and position of reference points in the field of view (FOV), two different phantoms were designed using MATLAB R2021a as shown in Figure 1. Each phantom was printed with a standard laser printer at 300 dpi and placed at the same position as the intensifying screen (isocenter). The image of the phantom was acquired using the same setup of RLI as shown in Figure 2.

The first reference phantom that has been investigated was a 10 × 10 mm^2^ square (Figure 1a). The transformation matrix *H* was calculated by manually choosing four corresponding points located at the corners of the reference and measured square phantom images.

As shown in Equation (4), it is possible to obtain a more robust image transformation by increasing the number of reference points between the reference and measured images (*N*). When more than 8 points are used, a pseudo‐inverse approach is applied to solving for the homography matrix. By taking into account the shape and dimensions of the CyberKnife® radiation fields, a second reference phantom, which we called “star,” was designed (Figure 1b). The star phantom was composed of five concentric circles with 24 cross markers distributed on the circumference of each circle and their diameter increased by 3.5 cm, respectively. Considering the largest field size in CyberKnife® (diameter = 6 cm) in order to optimize the image distortion correction, all the possible corresponding points (73 points) were taken into account to calculate the homography matrix. In this case the reference points of the star phantom were manually chosen at the center of each cross (Figure 1b).

In order to measure the accuracy of the image transformation procedure two *H* matrices obtained using the square and star reference phantoms were applied to the photographic image of a square as shown in Figure 4. The profiles of the distortion corrected square images were then compared with the reference (known) image.

**FIGURE 3:**
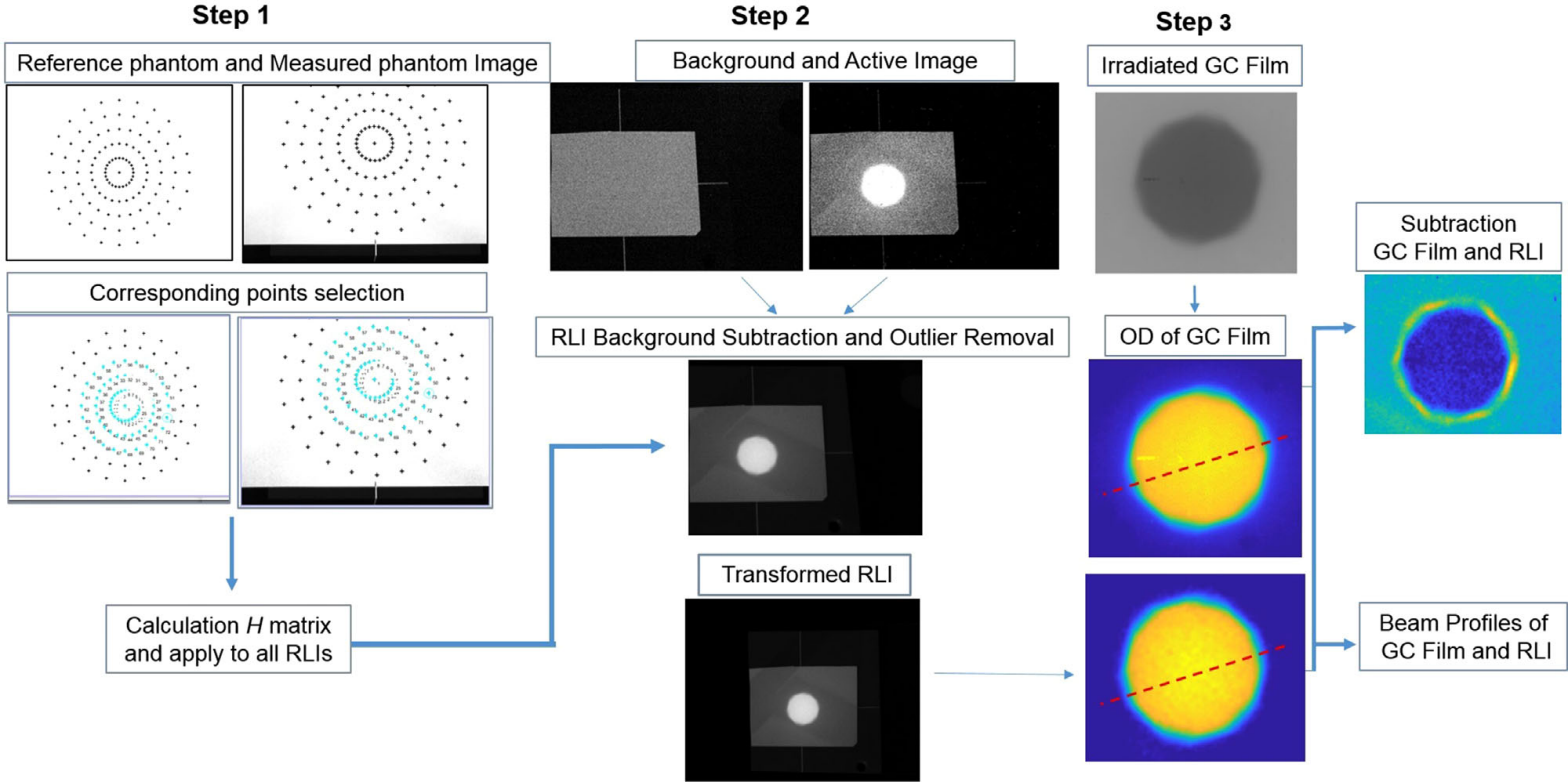
An overview of the image‐processing algorithm described in Sections 2.1 to 2.5. Step 1: *H* matrix is calculated using 73 corresponding points of the star phantom. Step 2: Spikes and Gaussian noise are removed from RLIs and background is subtracted. All the frames are then summed in order to obtain a cumulative RLI image. The *H* matrix is applied to the summed RLI image in order to remove the geometric distortion. Step 3: The beam profiles are extracted using the normalized RLIs and GC films and then compared.

**FIGURE 4:**
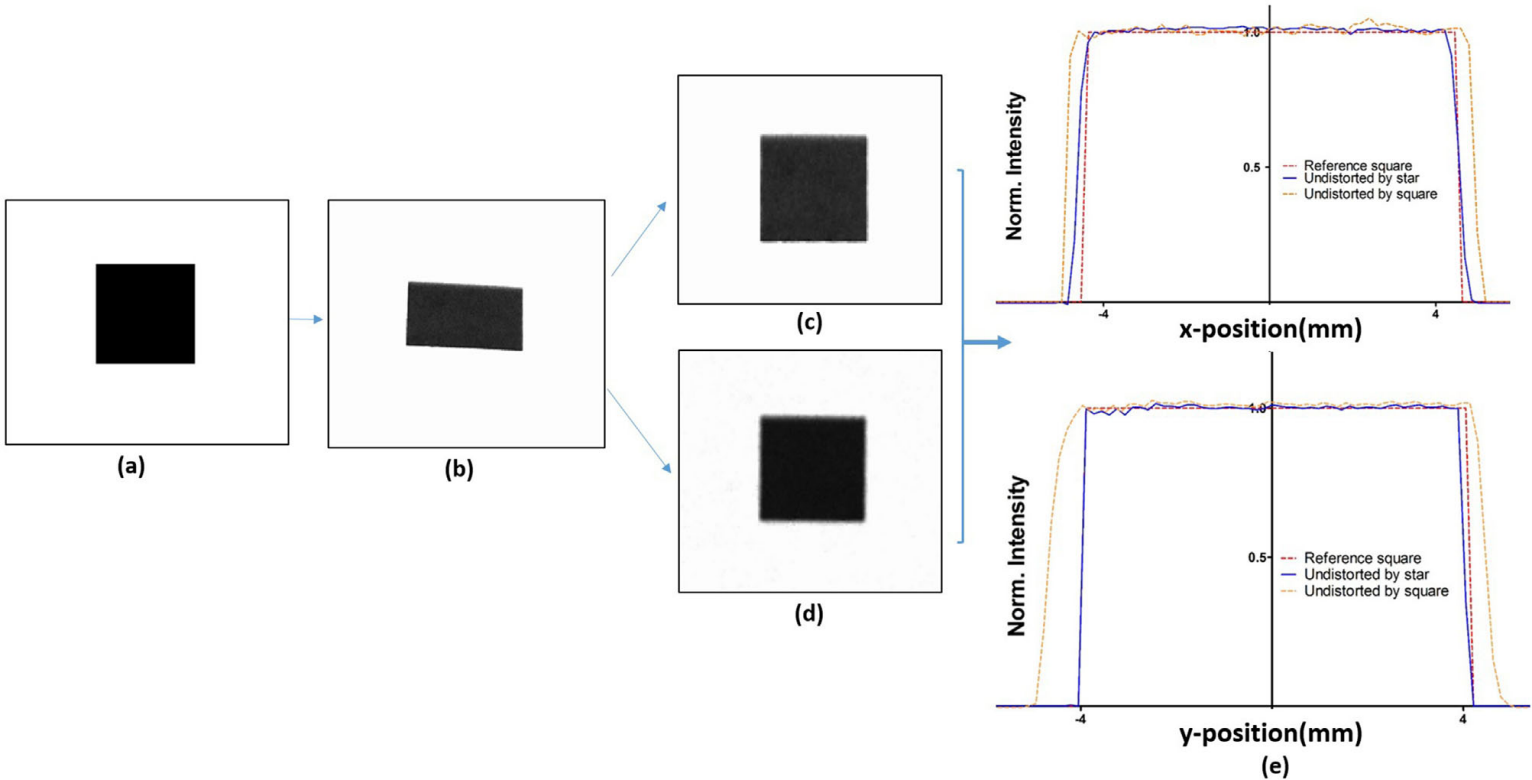
Improvement of perspective image distortion correction. (a) Reference square image, (b) measured square image using the proposed optical setup, (c) transformed images using the two *H* matrices obtained with the square phantom and (d) star phantom, (e) profiles of the transformed images with respect to the reference (known) image in both *x*‐ and *y*‐directions.

### 2.2 RLI system setup

As shown in Figure 2, our main goal was to investigate the performance of RLI as a 2D QA method for robotic radiosurgery. The RLI approach implemented in this work is based on a CMOS (the imaging source, DFK 33GX264) detector coupled with a thin (∼ 0.2 mm), flexible intensifying screen (MAMORAY Detail R screen, Agfa) for the detection and conversion of X‐rays in photons within the visible light range.^25^ Even if the intensifying screen is not optimized with respect to the CyberKnife® X‐rays mean energy, the emitted visible light signal is high and can be detected using a short exposure time. The visible photons emitted by the intensifying screen were collected using a lens and detected by the CMOS. The CMOS camera is composed of 2048 × 2048 pixels with a size of 3.45 μm. The intensifying screen (Gd2O2S:Tb) emits a light signal with an emission peak of 545 nm, which aligns well with the peak sensitivity of the CMOS camera.

Dose measurements using RLI were performed with the Cyberknife® system installed at our institute. The CMOS was mounted on a photographic tripod and coupled with an *F* = 1.4 , 8 mm C‐mount lens (Edmund Optics) facing the scintillation screen taped on the stereotactic dose verification phantom^30^ placed on the patient couch. The lens and the camera were at a distance of 105 cm from the isocenter with an angle between the lens optical axis and beam axis of approximately 15°. The center of the screen was placed at the isocenter while the SSD was set to 80 cm (Figure 2). The CMOS acquisition settings were gain = 22, brightness = 50, and exposure time = 0.02 s.

Irradiation was performed for 1 min using a 6 MV flattening‐filter free (FFF) beam at a dose rate of about 1000 monitor units per minute (MU min^−1^). The gantry was set to be perpendicular to the couch.

The scintillation screen was irradiated with radiation fields of different size using the fixed circular cones and dynamic variable‐aperture iris collimators. The RLI measurements were performed considering the following fields for each type of collimator: 5, 10, 40, and 60 mm. For each field size, a sequence of 120 RLI was acquired at a rate of 2 fps and saved for the analysis. We reduced the background light by switching off the ambient lights during image acquisition.

All the image processing procedures were performed using MATLAB R2021a as follows. Random spikes in the images, due to direct X‐rays interaction with the CMOS, were removed and replaced using the fill‐outliers function, then a 2D Gaussian filtering was applied to each image of the series to remove the Gaussian noise. Finally, the background was subtracted from RLIs and all the acquired frames were summed. More precisely the background was removed by taking the mean of a set of 10 images acquired before turning the beam on. All the analysis were performed on the summed image.

### 2.3 Field profiles

To transform the viewing angle of RLIs to a beam's eye view, the more accurate *H* matrix (see Section 2.1) was applied to the cumulative RLI images acquired as described in Section 2.2. Line profiles of the circle fields were extracted along the *x*‐ and *y*‐directions of the undistorted RLIs (see Figure 2) over the central row/column of pixels. In the case of the iris fields, because of polygonal field shape, the beam profiles were measured in the direction of the two sides facing each other as shown in Figure 3, step 3. The beam profiles were then compared with the profiles obtained using GC film by measuring the full width at half maximum (FWHM) calculated using spline interpolation.

### 2.4 GC films measurements

GC EBT3 (Ashland Inc., Covington, Kentucky) films were used as a gold standard for comparison with the RLI measurements. The GC film was positioned in the same location as the RLI with a 1 cm build‐up. Films were calibrated at SAD = 80 cm using different doses between 0 and 12 Gy.

In this work, films were irradiated as for RLI with about 1000 MU for 60 s using the same field sizes. GC films were then digitized 24 h after exposure at 16 bit and pixel size of 0.084 mm^2^, as routinely performed for periodic QA purposes.^31^ In order to calculate the optical density of GC film using MATLAB R2021a, the calibrated pixel value of the red channel of the corresponding RGB image was used.^32^ The FWHMs of all filed sizes were extracted and compared with RLIs. An overview of all the procedures was summarized in Figure 3.

### 2.5 RLI sensitivity to beam size variation

In order to investigate the sensitivity of the RLI method with respect to small geometrical variation of the beams, the source‐to‐surface distance (SSD) was slightly shifted in the positive *z*‐direction by 16 mm and 2.7 mm for circle 10 mm and 60 mm, respectively. These shifts in SSDs correspond to an FWHM increase of 0.2 mm.

For the iris field size constancy our tolerance level is 0.4 mm; for this reason we tested the sensitivity of the device to detect inferior errors; and we think that 0.2 was sufficient. The beam profiles were then measured using both the RLI and GC film.

### 2.6 Beam measurement reproducibility with RLI

The reproducibility of the entire procedure, in particular CMOS settings, reference image acquisition, and *H* matrix calculation, was investigated by measuring the beam FWHM and its standard deviation (SD) of the 60 mm circle field for three times on two consecutive days.

## 3 RESULTS

### 3.1 Perspective image distortion correction

Figure 4 shows two transformed images after applying the corresponding *H* matrices extracted from square and star reference phantoms.

The transformed images profiles in both *x*‐ and *y*‐directions were compared with the reference image as indicated in Figure 4e. The mean differences between the size of the reference and distortion corrected images in *x*‐ and *y*‐directions are 0.181 mm and 0.83 mm using the star phantom and square phantom, respectively.

### 3.2 Field profiles

**FIGURE 5:**
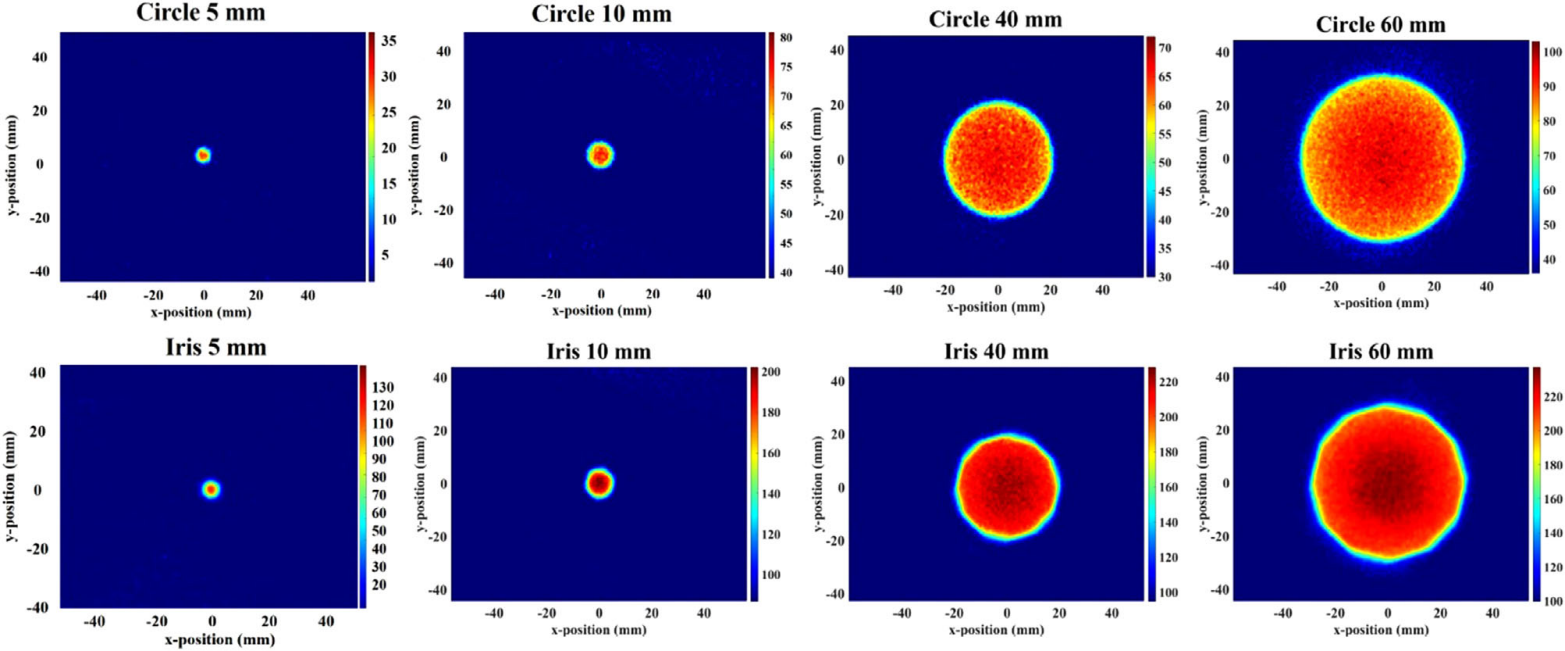
Perspective corrected RLI images for the different circle and iris field sizes.

Figure 5 shows the RLI for the eight tested field sizes corrected for perspective image distortion.

**FIGURE 6:**
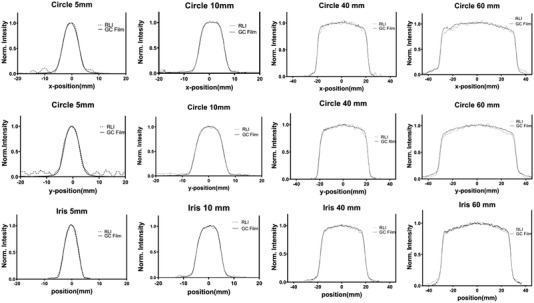
The plots show a comparison between the normalized beams profiles obtained with RLI and the corresponding profiles obtained from GC films for circle fields in both *x*‐ and *y*‐directions and for iris fields in the direction of the two facing sides as shown in Figure 3.

Beam profiles were measured for fixed circular cones collimators, the *x*‐ and *y*‐directions profiles are shown in Figure 6 and compared with the beam profile obtained using GC film measurements for each size. In the case of iris fields, the beam profiles were measured along the direction of the two sides facing each other (see Figure 6). As can be seen, differences between RLI and the GC film are only in the penumbra region.

In order to compare the noise of RLI and GC, we calculated the contrast to noise ratio (CNR) for the 60 mm circular and iris beams in a central 1 cm diameter region of interest (ROI). We found CNR values around 110–120 for CG and 80–100 for RLI.

Table 1 presents a comparison between the measured FWHM values using RLI and GC films. The average discrepancies of FWHM between RLI and GC film for circle fields are −0.0305 mm (−0.39%) and 0.0045 mm (0.037%) for *x*‐ and *y*‐directions, respectively. While, the average FWHM difference for the iris fields is −0.042 mm (0.146%). Of note, the maximum deviation was well below 0.1 mm.

**FIGURE 7:**
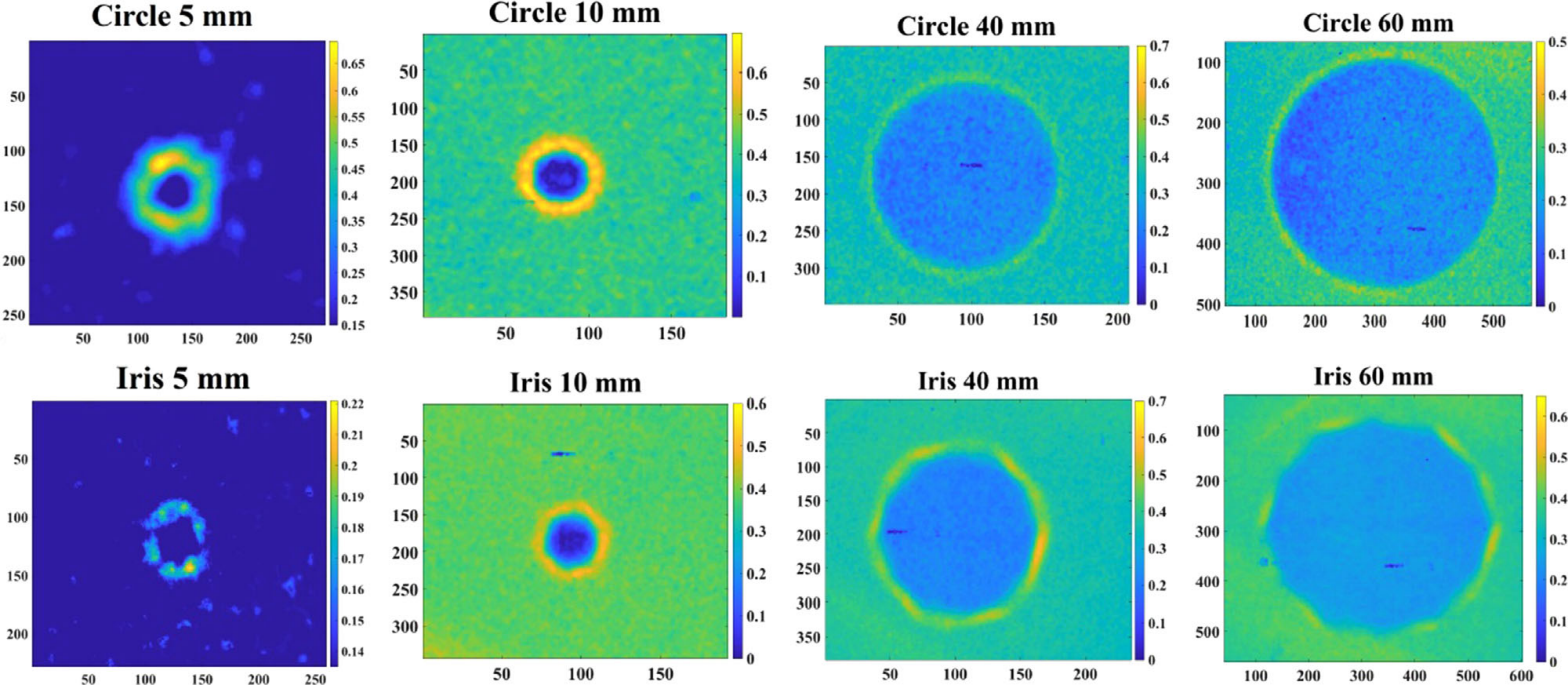
The images show the subtraction between the RLI and corresponding GC films. The main differences can be found in the penumbra region while differences are negligible in the central region.

RLI dosimetry was also compared with GC film by subtracting the two images as shown in Figure 7. As can be seen, the differences in the central region of the beam (above 50% of the dose) are negligible, while the main discrepancies are only in the penumbra region.

### 3.3 RLI sensitivity to beam size variation

Table 2 shows the known and measured field sizes using RLI and GC film, the SSD was shifted on purpose to increase the field size as much as 0.2 mm for both the circle 10 mm and 60‐mm beams. The RLI differences in the FWHM were equal to 0.246 mm for the 60 mm field and 0.317 mm for the 10 mm field. The FWHM differences measured with the GC films are 0.196 mm and 0.407 mm for field sizes 60 mm and 10 mm, respectively.

**FIGURE 8:**
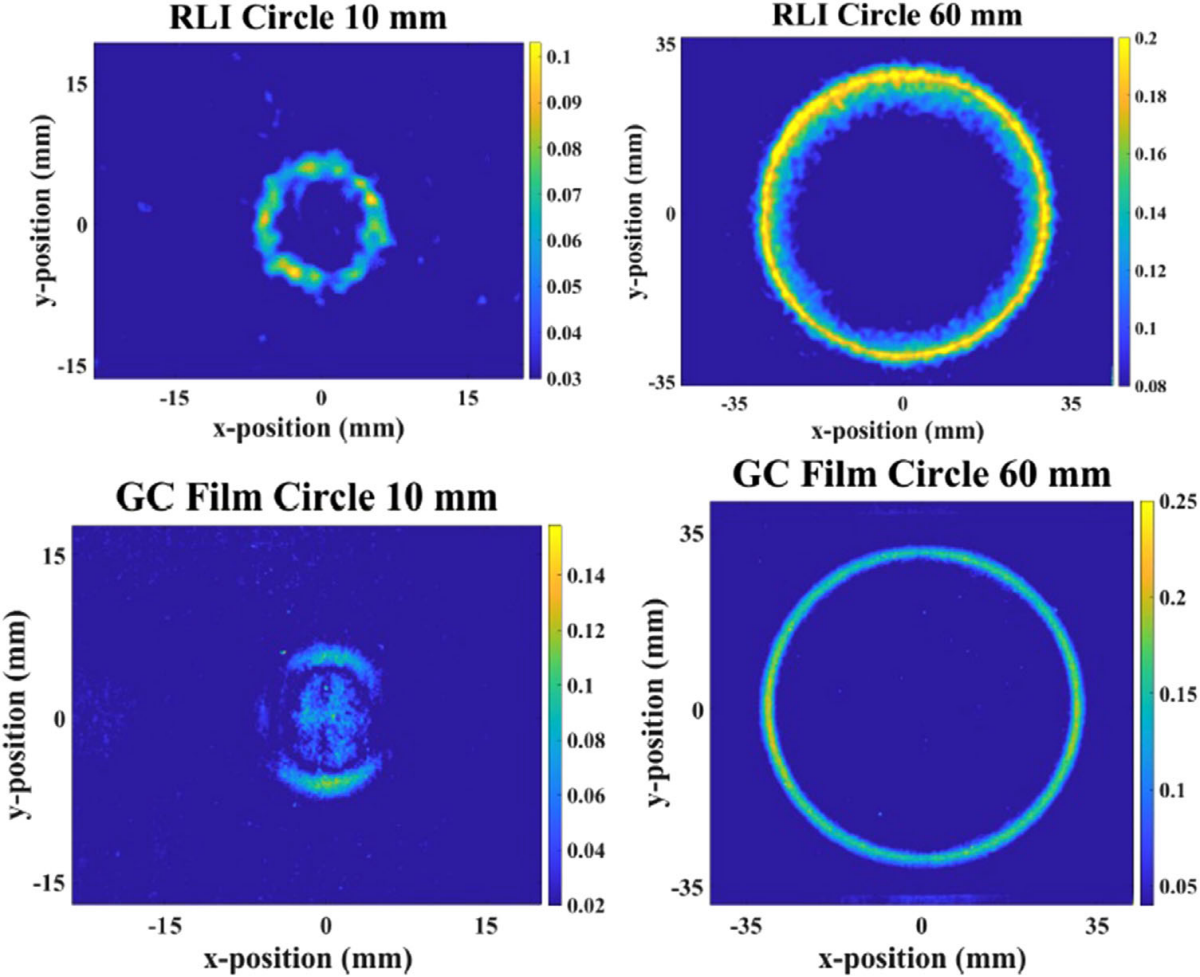
The images displays the differences due to small SSD shifting for both RLI and GC film. As can be seen, the sensitivity of the RLI approach allows the detection of small field size variations.

Figure 8 displays image differences between measurements with small SSD shift for both RLI and GC film. As can be seen the sensitivity of the RLI approach allows the detection of small field variations.

### 3.4 Beam measurement reproducibility with RLI

Beam profiles and FWHM were measured for fixed circular cones collimator 60 mm for three times on two consecutive days. The measured FWHMs are 60.997 mm, 60.912 mm, and 61.191 mm, respectively. The SD for these measurement is 0.114 mm. Thus, the average measured FWHM for field 60 mm is FWHM = 61.033 ± 0.114 mm.

## 4 DISCUSSION

This study describes the feasibility of the RLI approach based on a commercially available radioluminescence screen coupled with a CMOS camera to perform high‐resolution beam characterization for CyberKnife® routine QA procedures.

The performances of the RLI distortion correction procedure was investigated using a printed phantom with known shapes and dimensions. Figure 4 shows differences in the dimension after applying *H* matrices obtained using the square and star phantoms. Such differences not only reveal the importance of perspective distortion correction, but also emphasizes that the distortion correction can be significantly improved if the appropriate phantom and more reference points are considered. As presented in Figure 5, the image correction procedure is thus able to reduce perspective distortion in RLI measurements obtained using both circle and iris collimators of different size.

More importantly, the beam profiles obtained with RLI agreed quantitatively with the profiles obtained using GC film (gold standard) as shown in Figure 6. Besides Figure 6, a further 2D analysis presented in Figure 7 showed that dose differences in the central region are negligible and the main discrepancies are in the falloff regions. By looking at the plots in Figure 6, we can claim that the differences between RLI and GC in the penumbra region are relatively small for QA purposes. This applies to all the fields (from 5 mm up to 60 mm). The differences in the penumbra region might be due to light scattering within the scintillator screen and can be decreased by reducing the scintillator thickness. The build‐up material (1 cm) placed over the GC film could also lead to some difference in the penumbra region.

The data reported in Table 1 also show that the FWHM measurements have strong agreement with the GC film field for all the field sizes. The average (*x*‐ and *y*‐directions) discrepancies between RLI and GC film for all the circle and iris field sizes were −0.013 mm and −0.014 mm, respectively (< −0.07%). As already mentioned this accuracy was obtained by a more robust calculation of the *H* matrix based on the star phantom.

Considering the actual RLI pixel size of 0.162 mm, the system was expected to detect 0.2‐mm difference in the beam FWHM obtained by modifying the SSD. As presented in Table 2 and Figure 8, the RLI readout agrees with the expected differences in both field sizes 10 mm and 60 mm (average difference 0.28 mm).

As described in the previous section the RLI provides similar dose measurement with respect to the GC film, however had the advantage of instantaneous readout and simplified data collection. One of the future goal is to install the CMOS camera permanently on the head of CyberKnife® in order to reduce the setup time. In this case, if the SSD remains fixed, the *H* matrix can be calculated once and then applied to all RLI. In addition, the RLI system with a fixed geometry can be calibrated in order to obtain absolute dosimetry.

The effects of radiation damage to the optical system performance were not investigated directly. Nevertheless, it was estimated that a total dose of 100 Gy was delivered during the measurements without any significant degradation of the RLI images. It needs to be taken into account that the lens and the CMOS camera were not exposed with the primary beam, and were thus hit only by scattered radiation.

## 5 CONCLUSIONS

To the best of our knowledge, this study presents the first application of the RLI technique as a novel 2D detection system for robotic radiosurgery QA. This approach based on flexible scintillator screen and a CMOS detector yielded a fast readout measurements for CyberKnife® with good agreement with GC film. The sensitivity and sub‐mm spatial resolution of the system lead to a small deviation from the expected dose distribution profiles. These properties and the immediate availability of the data make RLI system a useful tool for robotic radiosurgery QA. The paper is mainly a proof of principle study focused on measuring cone and iris field size. We envision that the RLI approach could be used for other QA tests, like CAX‐laser coincidence checks, garden fence/picket fence, or other similar tests in order to verify multileaf collimator (MLC) positioning accuracy, symmetry/flatness constancy, and output factors. The same approach could be used for Automatic Quality Assurance Test (AQA) test, by using a different specific phantom, with a different beam path. The CMOS system could be included in the CyberKnife® head leading to a reduced setup time. Future work will mostly focus on this development and on a further optimization of RLI acquisition in particular by increasing the sampling rate.

In summary, RLI with CMOS camera demonstrated to be a promising approach for QA tests of CyberKnife®. Further investigation is warranted to replace all (or a large part of) tests usually performed with GC films and to extend it to dose distribution verification for the CyberKnife®.

## CONFLICTS OF INTEREST

The authors have no relevant conflicts of interest to disclose.

## Data Availability

The raw data supporting the conclusions of this paper will be made available by the authors, without undue reservation.
